# Personalization of Therapy in High-Grade Serous Tubo-Ovarian Cancer—The Possibility or the Necessity?

**DOI:** 10.3390/jpm14010049

**Published:** 2023-12-29

**Authors:** Jacek Wilczyński, Edyta Paradowska, Miłosz Wilczyński

**Affiliations:** 1 Department of Gynecological Surgery and Gynecological Oncology, Medical University of Lodz, 4 Kosciuszki Street, 90-419 Lodz, Poland; 2 Laboratory of Virology, Institute of Medical Biology of the Polish Academy of Sciences, 106 Lodowa Street, 93-232 Lodz, Poland; eparadow@cbm.pan.pl; 3 Department of Gynecological, Endoscopic and Oncological Surgery, Polish Mother’s Health Center—Research Institute, 281/289 Rzgowska Street, 93-338 Lodz, Poland; jrwil@wp.pl; 4 Department of Surgical and Endoscopic Gynecology, Medical University of Lodz, 4 Kosciuszki Street, 90-419 Lodz, Poland

**Keywords:** ovarian cancer, prediction, biomarkers, chemo-resistance, genomic signature, epigenetic signature, cancer subtypes

## Abstract

High-grade serous tubo-ovarian cancer (HGSTOC) is the most lethal tumor of the female genital tract. The foregoing therapy consists of cytoreduction followed by standard platinum/taxane chemotherapy; alternatively, for primary unresectable tumors, neo-adjuvant platinum/taxane chemotherapy followed by delayed interval cytoreduction. In patients with suboptimal surgery or advanced disease, different forms of targeted therapy have been accepted or tested in clinical trials. Studies on HGSTOC discovered its genetic and proteomic heterogeneity, epigenetic regulation, and the role of the tumor microenvironment. These findings turned attention to the fact that there are several distinct primary tumor subtypes of HGSTOC and the unique biology of primary, metastatic, and recurrent tumors may result in a differential drug response. This results in both chemo-refractoriness of some primary tumors and, what is significantly more frequent and destructive, secondary chemo-resistance of metastatic and recurrent HGSTOC tumors. Treatment possibilities for platinum-resistant disease include several chemotherapeutics with moderate activity and different targeted drugs with difficult tolerable effects. Therefore, the question appears as to why different subtypes of ovarian cancer are predominantly treated based on the same therapeutic schemes and not in an individualized way, adjusted to the biology of a specific tumor subtype and temporal moment of the disease. The paper reviews the genomic, mutational, and epigenetic signatures of HGSTOC subtypes and the tumor microenvironment. The clinical trials on personalized therapy and the overall results of a new, comprehensive approach to personalized therapy for ovarian cancer have been presented and discussed.

## 1. Introduction

High-grade serous tubo-ovarian cancer (HGSTOC) is the most lethal tumor of the female genital tract due to several reasons, mainly delayed diagnosis, high proliferative potential, and chemo-resistance followed by a high recurrence rate. Therefore, the 5-year survival in the advanced patient population (clinical stages III–IV) is invariably unsatisfactory ((data from the American Cancer Society 2020. https://www.cancer.org/cancer/ovarian-cancer/detection-diagnosis-staging/survival-rates.html) accessed on 25 August 2023)). Modern therapy consists of aggressive cytoreductive surgery followed by standard platinum/taxane-based chemotherapy or neo-adjuvant platinum/taxane chemotherapy followed by delayed interval cytoreduction in primary inoperable tumors. In the case of advanced primary or recurrent tumors, combined adjuvant therapy using the anti-VEGF (vascular-endothelial growth factor) humanized monoclonal antibody bevacizumab has been approved, resulting in only a moderate improvement in progression-free survival [1,2,3]. In the group of HRD-deficient patients whose tumors lack proficient homologous recombination repair mechanisms, the acceptable efficacy of the poly-ADP ribose polymerase (PARP) inhibitors in both adjuvant combined therapy and maintenance monotherapy has been shown [4,5]. The most destructive feature of HGSTOC is the chemo-refractoriness of primary tumors (which is not that frequent) or secondary chemo-resistance to standard platinum/taxane-based chemotherapy in advanced and recurrent cases (which is eventually observed in all recurrent or disseminated tumors) that follows initial chemo-sensitivity. To overcome chemo-resistance, several approaches to the therapy have been proposed, however, with very diverse efficacy and toxicity. The treatment possibilities for platinum-resistant disease consist of several non-platinum/taxane chemotherapeutics with moderate anti-tumor activity and different targeted drugs tested in a clinical trial setting, showing so far hardly acceptable effects. Recent studies on HGSTOC have discovered its genetic and proteomic heterogeneity, epigenetic regulation, and the modulating role of the tumor microenvironment. These findings turned attention to the fact that several different primary HGSTOC cancer subtypes exist and that primary, metastatic, and recurrent tumors are characterized by individual biology and thus a diverse drug response [6,7,8,9]. Therefore, the question appears as to why the different subtypes and temporal stages of HGSTOC ovarian cancer are predominantly treated based on the same therapeutic schemes rather than in an individualized way adjusted to the particular tumor subtype biology and temporal moment of the disease. The personalization of treatment should be understood as a proper treatment adjusted to the tumor type, tumor temporal heterogeneity, and genomic and/or metabolomic signature. Additionally, it should also mean that therapy fits key components of the tumor environment and the general and immunological status of the patient [10]. One of the most important tasks to personalize HGSTOC therapy is searching for individual tumor signatures that could differentiate therapy in a way that would enable the most effective tumor elimination. The new combinations of drugs are being tested in several clinical trials to overcome HGSTOC chemo-resistance and to better fit the tumor biology, but this is merely the dawn of the future therapy waiting behind the horizon [11,12,13,14,15,16]. The review presents the complex landscape of genomic, mutational, and epigenetic signatures of both HGSTOC subtypes and tumor microenvironments. Finally, the basic rudiments of a novel approach to the therapy of HGSTOC have been postulated.

### 1.1. Genomic Signatures of HGSTOC Cancer Cells

The first attempt to divide ovarian cancer tumors on the basis of genomic signatures was presented by Tothill et al. in 2008 [17]. The HGSTOC tumors were grouped into four subtypes: C1—high stromal response; C2—high immune signature; C4—low stromal response; and C5—mesenchymal, with a low immune signature. The C3 and C6 tumors were LGSOC and low-grade endometroid tumors, respectively. The C1 HGSTOC subtype indicated exclusively high expression of stroma-regulating genes and moderate to extensive desmoplasia. Desmoplasia was especially expressed in extra-ovarian (metastatic) peritoneal localizations and primary peritoneal HGSTOC tumors. Low numbers of intratumoral CD3+ T-lymphocytes were identified in the C1 subtype. Genes and signaling pathways associated with immune cells were found in the C2 subtype, with evidence of immune response represented by markers of T-cell activation and T-cell trafficking. Tumors from the C4 subtype also showed a high number of both intratumoral and stromal–associated T CD3+ cells and lower intensity of epithelial-mesenchymal transition (EMT). The C5 HGSTOC subtype was characterized by over-expression of genes engaged in mesenchymal development. The C5 subtype was further defined by the over-expression of proliferation and extracellular matrix–related genes and the low expression of immune response–regulating genes. Patients from the C1 and C5 subtypes showed poorer survival compared with other subtypes, and the C1 subtype was especially correlated with early relapse and short OS [17].

Next, the classic dualistic model of ovarian carcinogenesis was proposed in 2010 by Kurman and Shih and revised and expanded by them in 2016 [18,19]. In that model, ovarian cancer origin, biology, and genotype were defined and correlated with clinical outcomes. According to the model, ovarian cancer has been divided into type I tumors containing low-grade ovarian cancer (LGOC) of serous, mucinous, and endometroid histology with a better prognosis and relative chemo-sensitivity, and type II tumors of highly malignant and rapidly progressing high-grade ovarian cancer (HGOC) with a poor prognosis and high mortality rate, and good chemo-sensitivity, which, however, turns quickly into secondary resistance. Type I tumors are associated with relative genetic stability, while type II tumors have high chromosomal instability, defective homologous recombination repair, and are characterized mostly by *TP53*, *BRCA1*, *BRCA2*, *RB1*, and catenin beta 1 (*CTNNB1*) mutations [18,20,21,22,23].

The different classification of HGSTOC (although similar to that proposed by Tothill et al.) was presented by The Cancer Genome Atlas Research (TCGA) Network and was based on both gene expression pattern and histological structure; it divided ovarian cancer into four subtypes: mesenchymal (28%), immunoreactive (21%), proliferative (20%), and differentiated (17%). The mesenchymal subtype showed desmoplasia and an invasive mesenchymal gene expression pattern, with high expression of markers of increased stromal components such as those found in myofibroblasts and microvascular pericytes. The proliferative subtype showed limited inflammatory infiltration and activation of signaling pathways for stemness, low expression of ovarian tumor markers, and high expression of proliferation markers. Both subtypes presented an unfavorable prognosis. The immunoreactive subtype was characterized by extensive T-cell tumor infiltration and Toll-like receptor signaling, as well as the upregulated expression of T-cell chemokine ligands. It was characterized by a better prognosis. Differentiated subtype has a gene pattern resembling that of serous borderline tumors and were associated with high expression of tumor markers and with an expression of the secretory fallopian tube maker, suggesting a more mature stage of development. It had an intermediate prognosis [24,25,26].

Another TCGA-based study developed subtype and survival gene expression signatures, which, when combined, provided a prognostic model of HGSTOC classification, named “Classification of Ovarian Cancer” (CLOVAR). The HGSTOC subtypes were named analogically to the subtypes of the original TCGA study, as CLOVAR Immunoreactive, CLOVAR Mesenchymal, CLOVAR Proliferative, and CLOVAR Differentiated subtypes. Within each of the CLOVAR subtypes the CLOVAR good and CLOVAR poor prognosis signatures were used. The worst outcome was found in patients with CLOVAR Mesenchymal/CLOVAR poor prognosis tumors (23% of HGSTOC cases) [27].

Integrative analysis of the tumor genome identified three novel ovarian cancer subtypes named tumor-enriched, immune-enriched, and mixed. The tumor-enriched subtype was characterized by high expression of keratin cancer oncogenes and low expression of the immune regulatory molecule CD45 and the immune checkpoint protein PD-1, which are regulators of T- and B-cell antigen receptor signaling and T-cell apoptosis, respectively. Unfavorable, the immune-enhanced subtype showed the high expression of immune CD45 and PD-1 and its ligand PD-L1, accompanied by low expression of tumor oncogenes. The mixed subtype had a mixed expression pattern. The meaning of these subtypes for therapy implies that tumor-enriched tumors should be treated with tumor-killing therapy, while immune-enriched tumors with immunotherapy or a mixture of both approaches [28].

Another study investigated the molecular characterization of platinum-refractory and platinum-resistant ovarian tumors. There were three clusters identified—cluster 1 with an over-representation of growth factor signaling pathways, cluster 2 with a representation of pathways regulating cell survival in hypoxic conditions and senescence, and cluster 3 related to cellular senescence. These differences, however, did not significantly affect patients’ survival, which was similar in all clusters. However, the knowledge about the differences could influence the therapeutic approach to the tumors. A possible treatment of choice for cluster 1 could be tyrosine kinase or angiokinase inhibitors. Clinical trials with the use of nintedanib and cediranib confirmed the prolongation of PFS in patients treated with a combination of these drugs with platinum chemotherapy [8,29]. Analogically, the potential therapy for cluster 3 could be the use of deacetylase inhibitors [30]. Cluster 2 could theoretically respond to mTOR inhibitors; however, the broad spectrum of responses to this kind of therapy needs further investigation [31].

Using single-cell RNA sequencing, Shih et al. were able to identify distinct cell populations correlated to different tumor grades and their primary or metastatic origin [32]. The hierarchical clustering of cells from all patients resulted in three main groups: HGSTOC primary tumor, HGSTOC metastatic tumor, and LGSOC tumor. Primary HGSTOC samples were predominantly epithelial cells (68% epithelial cells versus 11% lymphocytes), whereas corresponding metastatic samples were predominantly immune cells (66% lymphocytes versus 10% epithelial cells) with fewer amounts of epithelial cells, fibroblasts, and stromal cells. HGSTOC epithelial cells showed high expression of genes responsible for promoting microtubule aggregation, cell proliferation, and mitosis, as well as ciliogenesis. Tumor fibroblasts and stromal cells showed up-regulation of collagen and collagen-supporting genes, as well as, metalloproteinase-related genes. Compared to fibroblasts derived from the primary tumor, metastatic fibroblasts express proteins that stimulate tumor growth, angiogenesis, and inflammation. The cell cluster patterns corresponded to the TGCA subtypes [24,25,26] in that way that the LGSOC epithelial cells were identified as the differentiated subtype, primary and metastatic cells of myeloid lineage as the immune responsive subtype, and primary fibroblasts, metastatic fibroblasts, and cancer stromal cells as the mesenchymal subtype [32].

Another single-cell transcriptome study revealed the heterogeneity of HGSTOC, which was found to be composed of several cell clusters, similar to those described in the Shih et al. study. Tumor epithelial cells were enriched in EpCAM and keratins, as well as markers of the fallopian tube epithelium. Stromal markers, including decorin and collagen, were highly expressed in fibroblast cell clusters that exhibited cancer-associated fibroblast (CAF) markers. Immune cells were divided into three distinct clusters, including T lymphocytes, B lymphocytes, and macrophages [33]. The cluster of epithelial HGSTOC cells was further subdivided into five subtypes. The first one, called EC1, showed gene enrichment for glycolysis/gluconeogenesis, and ECM-receptor interactions. The EC2 subtype expressed genes involved in the cytokine-cytokine receptor interaction, neuroactive-related pathways, and ciliated epithelial markers, suggesting the origin of EC2 from the tube epithelium. The expression of the ciliated epithelial markers diminished during the process of metastasis, although they were over-expressed in EC2 of primary tumor cells, and the loss of strong expression of these markers predicted a worse prognosis in HGSTOC. The EC3 subtype showed over-expression of genes associated with nucleotide and amino acid metabolism and in the function of ABC transporters, suggesting a potential to be a drug-resistant subtype. The next EC4 subtype was characterized by immune response-related pathways and the complement cascade, thus indicating the activity of EC4 cells in the immune response. The last EC5 subtype displayed gene enrichment for pathways associated with the cell cycle, DNA replication, DNA repair, and drug metabolism. The chemo-resistance-controlling genes were strongly represented in the EC5 population, together with homologous recombination-associated genes, indicating that EC5 cells could be resistant to therapy, especially with PARP inhibitors [33]. The analysis of gene regulatory networks showed that in epithelial clusters the highest regulatory activity had transcription factors viewed as tumor promoters, including JUN. Observations suggested that JUN-dependent signaling pathways are one of the key promoters of ovarian carcinogenesis. The inhibition of JUN-signaling by the T-5224 inhibitor significantly abrogated HGSTOC survival and proliferation in vitro [33].

The single-cell RNA sequencing analysis performed by Li et al. [34] on HGSTOC cells and compared to the TCGA database showed that identified marker genes overlapped with differentially expressed genes in TCGA samples and that low expression of genes engaged in histone binding activity, response towards interferons, regulation of cell cycle and proliferation, ubiquitination, transfer of reducing equivalents from the physiological electron donor NADH, as well as cell growth arrest function, affected both OS and PFS of HGSTOC patients [34].

Integrated bioinformatics analysis of ovarian cancer and normal tissues from the Gene Expression Omnibus (GEO) showed differentially expressed genes (DEGs) between ovarian cancer and normal controls, and from among them, ten hub genes influencing unfavorably patients’ survival were identified. This gene signature included genes regulating kinesins (microtubule-based proteins that generate movement along microtubules), cell division cycle regulators, topoisomerase, enzyme reductase (which promotes proliferation and EMT through the JAK2/STAT3 signaling pathway), thymidylate synthetase (which plays an important role in DNA biosynthesis and repair), survivin (a regulator of apoptosis), kinase regulating the spindle checkpoint and chromosome segregation, and FOXM1, a transcription factor regulating cell cycle progression. Among them, thymidylate synthetase and survivin were indicated as potential drug targets due to their multiple downstream effects [35].

The next set of eight DEG genes was established to build the prognostic model and predict platinum therapy sensitivity in ovarian cancer. The up-regulation of four genes regulating: poly(A)-specific ribonuclease activity, cell-cell adhesion activity, protein in the voltage-dependent calcium channel complex, and a ligand of the transforming growth factor-beta (which binds to various TGF-beta receptors leading to regulation of gene expression) was related to an unfavorable outcome, whereas high expression of genes responsible for cell-cell junction, transmembrane glucose transporting, and synemin, which enables cell response against mechanical stress and maintains structural integrity in the cells, was associated with a better prognosis. All these genes were connected to different aspects of cancer growth and their changed expression was described in several cancers [36].

The characterization of metabolism-associated molecular subtypes in ovarian cancer enabled the identification of three subtypes (C1, C2, and C3). The C1 subtype showed decreased immune-cell infiltration and expression of immune checkpoint genes, the lowest incidence of *BRCA* mutation, and an optimal response to bevacizumab. The C2 subtype displayed the worst prognosis, up-regulated immune-cell infiltration and expression of immune checkpoint genes, and a suboptimal response to bevacizumab. The C3 subtype had an intermediate immune status, the highest incidence of *BRCA* mutations, and a secondary optimal response to bevacizumab [37].

The integrated molecular profiling of ascites and primary tumor cells, ovarian cancer stem cells (OCSCs), and patient-derived xenografted mouse tumors (PDXs) enabled the construction of three Quantitative Traits (QT) signatures. The QT-A was composed of genes involved in HGSTOC initiation expressed in PDXs and OCSCs, related to cell proliferation and tumor development. The QT-B contained genes involved in tumor-host interaction and dissemination, which were highly expressed in malignant ascites and inflammatory environments. It was dominated by genes regulating the inflammatory response, NF-*ĸ*B activity, EGF signaling, and hypoxia. Finally, QT-C with genes involved in mesenchymal-like and non-proliferating OCSCs-like cells [38]. It was noticed that the QT-A signature was predominant in the proliferative HGSTOC subtype, QT-B in the inflammatory subtype, and QT-C in the mesenchymal subtype, respectively [38].

Patients with HGSTOC demonstrated different copy-number signatures of the tumors depending on a combination of mutational processes. Signature 1 correlated with platinum-resistant relapse and poor OS. Signature 2 also correlated with poor OS and was characterized by a high number of breakpoints and single copy-number changes. Signature 3 was the result of *BRCA1/2*-related homologous recombination deficiency (HRD) and was associated with the diploid and single copy changes and favorable OS. The next signature 4 was the consequence of whole genome duplication. It was characterized by multiple copy-number changes. Signature 5 was characterized by subclonal copy-number changes. Signature 6 resulted from focal amplification produced by the loss of the cell cycle control. For signatures 4–6, the link with survival was uncertain. The last one, signature 7, was caused by non-*BRCA1/2*-related HRD and was characterized by different distributed copy-number changes and breaks. It was correlated with a favorable OS [39].

### 1.2. Mutational Signatures of HGSTOC Cancer Cells

The most abundant genetic alteration found in almost all HGSTOC tumors is the *TP53* mutation. The landscape of other mutations is characterized by the presence of different, less frequent mutations. The most studied *BRCA1/2* germline and somatic mutations occur in 9% and 3% of tumors, respectively. In 2–6% of HGSTOC tumors, the mutations of tumor suppressor genes, cell-cell interaction, and cellular morphology-regulating genes, as well as genes regulating the cell cycle, have been confirmed. Some less frequent mutations in proto-oncogene B-raf (*BRAF*), phosphatidylinositol-4,5-bisphosphate 3-kinase (*PIK3CA*), Kirsten rat sarcoma virus (*KRAS*), and neuroblastoma RAS viral oncogene homolog (*NRAS*) genes have been observed, as well as copy number alterations (CNAs) in the form of amplifications and deletions were found. The components of RB1, PIK3/RAS, NOTCH, and FOXM1 signaling pathways were mutated or aberrantly methylated, and together with homologous recombination defects were found in about 50% of HGSTOC tumors [40]. The whole genome sequencing studies combined with transcriptome and methylene analysis confirmed the prevalence of TP53 mutations and *BRCA1* promoter methylation in about 50% of HGSTOC tumors, as well as gene breakage in tumor suppressors like *RB1*, *NF1*, *RAD51B*, and *PTEN* and *CCNE1* amplification [22,41]. In another study, a serious homologous recombination (HR) dysfunction in HGSTOC was further confirmed, although the particular rates of mutations found differed slightly. Both germ-line and somatic mutations of *BRCA1/2* involved about 20% of patients, while an epigenetic silencing of *BRCA1* by hypermethylation involved about 10% of patients, respectively. Inactivation by deletion, mutation, or hypermethylation of other genes was also noticed, including *ATM*, *ATR*, *RAD51C*, *PTEN* (about 10% of patients), and Fanconi anemia member genes (about 5% of patients). Amplification of EMSY transcriptional repressor BRCA2-interacting—*EMSY* was also shown in 8% of patients. Collectively, at least 50% of HGSTOCs are thought to have HR pathway defects [24]. Approximately 30% of HGSC tumors have alterations in genes involved in *RB*-mediated DNA repair and cell cycle control, including amplification of *CCNE1* (20% of patients), loss of *RB1* (10% of patients), or gain of retinoblastoma-binding protein 8—*RBBP8* (4% of patients) [42].

The particular signature of HGSTOC is connected to the reactivity of some anti-cancer drugs. The observed mutual exclusivity of *CCNE1* amplification and *BRCA1/2* loss in HGSTOC explained the insensitivity of *CCNE1*-amplified tumors to platinum and suggested that these patients are unlikely to respond to PARP inhibitors, however, they could respond to proteasome inhibitor bortezomib [43]. The disturbed expression of several genes was associated with the progression of chemo-resistance to paclitaxel and carboplatin [44]. The knockdown of *RAD50* or the presence of *MYC* amplifications in HGSTOC cell lines was correlated with sensitivity to PARP inhibitors. In HGSTOC tumors with alterations of receptor tyrosine-protein kinase ERBB2 pathway members encoding genes, the combined treatment with platinum and anti-HER2 drugs showed better results [45]. Mutations of *KRAS*, *EGFR*, and protein kinase C-alpha (*PKC-α*) characterized the MEK inhibitor-sensitive and resistant cell lines, respectively. The combination of MEK inhibitors with EGFR inhibitors showed better results in resistant cell lines [46].

It was found that some mutations in patients with HGSTOC were already present in premalignant lesions of the fallopian tube epithelium, which confirms the dualistic model of ovarian cancer. Mutations in the repressor region of the key stem cell differentiation gene *SOX2* have been shown to be mutated in ovarian cancer patients, even in apparently normal tube epithelium. In superficial tubal intraepithelial cancer (STIC), which is an accepted progenitor lesion for HGSTOC, mutations in the somatic *TP53* tumor suppressor gene constitute the so-called “p53 signature”, which unexpectedly was also confirmed in the benign tubal epithelium of *BRCA* mutation carriers and normal controls. However, in the presence of STIC, the “p53 signature” was more abundant, multifocal, and predominant in the fimbriae (80–100%). STICs and their associated HGSTOCs shared identical mutations [47,48]; however, this was not confirmed in another study [49]. Expression of stem cell markers ALDH1 and/or SOX2 shows increased frequency in HGSTOC compared to low-grade LGSOC and borderline tumors, supporting the concept that stem cell markers play distinct biological roles in low- and high-grade serous neoplasia of the ovary [50].

### 1.3. Epigenomic Signatures of HGSTOC Cancer Cells

Analysis of immune-related long-non-coding (lnc) RNAs revealed seven pairs of lncRNAs having the potential to divide the population of ovarian cancer patients into high-risk and low-risk groups characterized by a shorter or longer OS, respectively. The high-risk group also showed correlations with clinical parameters of tumor advancement and with immune cell infiltrate, supporting the data from genomic studies in HGSTOC. High-risk scores were positively correlated with abundant infiltration of neutrophils, macrophages, cancer-associated fibroblasts, T cells, and mast cells. Moreover, co-stimulatory immune regulators and activation proteins were significantly lower, while suppressors of immune activation were higher in the high-risk group, respectively [51]. This means that the studied lncRNA paired signatures are capable of recognizing patients with compromised anti-tumor immune responses. Moreover, the comparison between ovarian cancer and normal ovarian samples revealed the presence of five differentially expressed lncRNAs, which were considered protective or risk factors. Based on the known function and regulatory pathways of those lncRNAs, five candidate small-molecule drugs (thioridazine, trifluoperazine, loperamide, LY294002, and puromycin) were predicted using bioinformatics analysis [52].

Integrated analysis of gene expression and DNA methylation signatures in ovarian cancer revealed several dozen both hypomethylated and hypermethylated genes involved in integrin/cadherin signaling pathways, amino acid biosynthesis, endocrine resistance, apoptosis, focal adhesion, and cellular senescence. Four genes, namely *TNF*, *ESR1*, *MUC1*, and *FOXO1*, can function as targets for epigenetic therapy and have been correlated with patients’ prognosis [53]. DNA methylation status is related to ovarian cancer pathophysiology. Analysis of methylation patterns identified six molecular subtypes, of which cluster 2 had the highest methylation level and demonstrated the best prognosis, while clusters 4 and 5 had significantly lower methylation levels compared to the other subtypes, were connected with the HGSTOC, advanced tumors, and demonstrated very poor prognosis. Analysis showed that DNA hypomethylation was significantly worse than hypermethylation [54]. The genomic, mutational, and epigenetic signatures of HGSTOC are presented in Table 1.

## 2. Genomic, Mutational and Epigenomic Signatures of HGSTOC Tumor Microenvironment

### 2.1. Cancer-Associated Fibroblasts (CAFs)

In addition to the identification of HGSTOC epithelial cell subtypes (EC1–EC5—described above), Hao et al. [33] recognized five HGSTOC-isolated fibroblast cell subtypes. The FC1 subtype showed gene enrichment for lipid and steroid metabolism. The FC2 subtype exhibited a preference for genes involved in glycolysis/gluconeogenesis, oxidative phosphorylation, and DNA repair pathways. Moreover, the FC2 population showed expression of tumor epithelial markers and markers of EMT. These cells represent the highly aggressive phenotype of CAFs enhancing tumor chemo-resistance. The FC3 cells were enriched in the genes responsible for the immune response-related pathways. In the FC4 subtype, the most prominent over-expression of angiogenesis-related genes was observed. The next FC5 subtype showed high expression of genes regulating lipid metabolic pathways, extracellular matrix signaling, and cellular stemness [33]. The importance of interactions between cancer cells and CAFs was represented by the expression of relatively high levels of epithelial and fibroblast growth factor receptors on cancer cells and their corresponding ligands on the CAF cells. Some receptor-ligand interactions were expressed at higher levels in metastatic compared to primary tumors, emphasizing their importance in cancer progression.

The study performed by Givel et al. [55] identified four different CAF subsets named CAF-S1 to CAF-S4. It was shown that HGSTOC of the mesenchymal subtype, defined by stromal gene signatures and poor survival, had high numbers of CAF-S1 cells that attract and maintain an immunosuppressive infiltration of Treg CD25+ FoxP3+ T lymphocytes through the expression of CXCL12*β* and miR-141/200a dependent-mechanism [55].

The study of the immunological profile of HGSTOC identified a novel immune group of HGSTOC with high infiltration of immune cells, lower chromosomal aberrations, increased neo-antigens, high tumor mutation burden, and microsatellite instability. Tumors belonging to the immune class showed enrichment of immune cell signatures, like T cells, B cells, cytotoxic cells, tertiary lymphoid structures (TLS), macrophages, as well as NK cells and PD-1 signaling. According to the tumor microenvironment, the immune group was divided into two microenvironment-based subsets, namely activated-immune and CAFs-immune subtypes. Both subtypes exhibited high expression of immune molecules; however, the activated-immune subtype showed anti-tumor features exemplified by enrichment of IFN signatures, active immune response, and better prognosis. The CAFs-immune subtype was characterized by tumor-promoting signals like activated stroma, M2 macrophages, WNT/TGF-*β* signaling pathway, and a poor prognosis. The activated-immune subtype was more likely than the CAFs-immune subtype to respond to checkpoint blockade immunotherapy [56].

Similar results were obtained in another study. The CAF content was described as the CAF-score. Analysis of immune cell infiltration showed that the infiltration of memory B cells, T-helper cells, T regulatory cells, activated natural killer cells, and dendritic cells was higher, while the infiltration of memory CD4 T cells, M2 macrophages, and neutrophils was significantly lower in tumors with a low CAF-score. A low CAF-score indicated a better prognosis and identified a group of patients with more immunogenic tumors who may be good candidates for immune checkpoint inhibitor therapy [57].

Transcriptome profiles from ovarian cancer CAFs identified two distinct subtypes of CAFs in HGSTOC tumors, including CAF-N and CAF-C. The CAF-C subtype was characterized by more aggressive behavior and a worse prognosis. Therapeutic use of calcitriol in tumor xenograft mice was able to inhibit Smad signaling in CAF-C cells and prolong median mouse survival [58].

### 2.2. Tumor Microenvironment Different Cell Populations

The study performed by Olbrecht et al. [59] showed HGSTOC cell clusters originating from ovarian, peritoneal, and omental tissue which were divided into 8 major cell types, including epithelial cancer cells, myeloid cells, dendritic cells (DCs), T cells (TCs), B cells (BCs), fibroblasts (FBs), endothelial cells (ECs) and ovarian stromal cells (OSCs). Based on differential gene expression analysis, cells isolated from omental implants were identified as Langerhans-like dendritic cells and lipid-associated M2 macrophages. In several tumor clusters, the genes of fallopian tube secretory cells were over-expressed, confirming the fimbrial origin of HGSTOC tumors [59,60]. The keratin-17 gene-positive cancer cells were represented in both the primary and metastatic localizations indicating that tumor cells at different places show similar transcriptomic profiles, and the observed heterogeneity should be attributed mostly to the differences in stromal cells. Some clusters had prognostic relevance. Mesothelial cells showed active EMT and activation of the IL6/STAT3 signaling pathway, analogically to the fibroblast cluster from the Izar et al. study [61], and were correlated to tumor growth and chemo-resistance. Myofibroblasts were engaged in hypoxia-mediated EMT, active TGF-*β*-pathway, and promotion of metastases. Cancer-associated fibroblasts were TGF-*β*-dependent and enhanced tumor growth, EMT, metastases, and platinum-resistance. Lymphatic endothelial cells were responsible for the lymphatic spread of metastases. The next prognostic cluster was represented by BMP and activin membrane-bound inhibitor homolog (BAMBI)-expressing tumor cells. Over-expression of BAMBI was observed in recurrent, but not in primary HGSTOC tumors, and was supposed to be one of the markers of platinum-resistance. The cluster represented by plasma cells was a good prognostic marker of an immune-active anti-tumor environment [59].

### 2.3. Tumor Microenvironment Immune Cell Populations

The study of immune-related gene pairs seems to confirm these observations and enables to differentiation of ovarian cancer patients into high- and low-risk groups according to the clinical outcome. The Toll-like receptor and chemokine signaling pathways were negatively correlated with the risk scores, indicating that the low-risk group tumors had immune infiltration of higher activation status. Contrarily, the p53 signaling and apoptosis pathways had a positive correlation with the risk scores. In addition, the authors also found that the best prognosis was obtained in the immunoreactive, while the worst prognosis was in the mesenchymal ovarian cancer subtype, respectively [62].

Bioinformatics analysis of the expression of the C-X-C motif chemokine receptors (CXCRs) in ovarian cancer indicated higher expression of CXCR3, -4, -7 mRNA and different expression of CXCR1, -2, -3, -4, -7 mRNA in different pathological types of ovarian tumors. High CXCR7 mRNA expression and low CXCR5, -6 expression were associated with unfavorable OS, while high CXCR4, -7 expression and low CXCR5, -6 expression were associated with a decrease of PFS [63]. The role of CXCRs is multifunctional. The CXCR1/CXCR2 are the receptors for IL-8, which is over-expressed in ovarian cancer patients both in serum and ascites [64]. CXCR3 is expressed on activated T, B, and NK cells and mediates ascites-directed tumor cell migration. Is correlated with poor survival in several cancers including ovarian cancer [65]. Over-expressed CXCR4 enhances proliferation and invasion of cancer cells through positive regulation of EMT via CXCR4/CXCL12 signaling, stimulates cellular stemness and chemo-resistance, and in consequence worsens PFS in ovarian cancer patients [66,67]. Similarly, CXCR5/CXCL13 signaling regulates EMT and negatively correlates to OS and PFS in ovarian cancer [63,68]. The CXCR6/CXCL16 signaling promotes cancer growth by activation of the PI3K/AKT pathway and is associated with TAMs function, expression of TNF-*α*, and docetaxel-resistance [69]. The CXCR7 has a high affinity to CXCL12 and transports signal for metalloproteinase-9 over-expression, as well as through CXCR7/CCL19 signaling up-regulates mesenchymal phenotype, thus stimulating metastases [70,71]. The CXCR3/4/7 could be potential therapeutic targets in ovarian cancer. The CXCR4 inhibitor AMD3100 was successfully used in in vitro studies and mouse xenograft models of ovarian cancer to restore taxol chemo-sensitivity and prolong survival [72,73].

An immune-related lncRNA pairing model for predicting tumor immune infiltration and cancer prognosis was constructed based on the expression of seven lncRNA pairs. The high-risk lncRNA signatures correlated with tumor infiltration with macrophages, neutrophils, T and mast cells, and CAFs. Moreover, the tumors with high-risk signatures showed low expression of immune checkpoint-related genes [51].

### 2.4. Tumor Microenvironment Cell Populations in Ascites

Izar et al. [61] analyzed cellular populations of HGSTOC ascites using single-cell RNA-sequencing and demonstrated significant variability in cellular states among malignant and non-malignant cells. The identified populations were composed of epithelial cells, cancer-associated fibroblasts, dendritic cells, B cells, T cells, and erythrocytes. Among CAFs, there were identified distinct cell states, including sub-populations with expression of immune-related genes, such as complement factors and cytokines. Among macrophages generally, two groups were identified. Group 1 cells co-expressed several genes identified as markers of M1-type macrophages and suppressors of M2 differentiation, whereas Group 2 cells expressed genes regulating M2 differentiation. Authors concluded that the previously described “immunoreactive” and “mesenchymal” subtypes of HGSTOC, reflected the abundance of immune infiltrates and fibroblasts rather than distinct subsets of malignant cells [61].

The cellular components of ascites include either single cells or cell aggregates called spheroids. The single-cell population was composed mostly of immune cells, some tumor cells, and CD90+ mesenchymal-like cells. The spheroid population was composed almost exclusively of EpCAM+ and CD24+ tumor cells. Ascitic spheroids showed, compared to the primary or metastatic ovarian cancer cells, significantly up-regulated genes related to the oxidative phosphorylation pathways, chemo-resistance, encoding glycosylation enzymes, and transcription factors. Over-expressed protein-glutamine gamma-glutamyltransferase K gene (*TGM1)* and up-regulated heat shock proteins in the spheroid cells promoted their stemness and chemo-resistance, whereas proteins with mechanical barrier function, supporting cellular aggregation (plakophillin, periplakin, claudin, filaggrin) could protect cancer cells from immune recognition and destruction [74]. Spheroids contain M2-type tumor-associated macrophages (TAMs) and also CAFs which together enhance the aggregation and adhesion of these tumor cell aggregates [75]. The spheroids indicated down-regulation of angiogenesis and extra-cellular structure organization pathways and significant up-regulation of the mitochondrial oxidative phosphorylation (OXPHOS) pathway. This is typical for quiescent cells with low proliferation profile and low chemo-sensitivity, as are spheroids [76,77]. Drugs inhibiting the OXPHOS pathway could be considered in ovarian cancer therapy. Potential OXPHOS inhibitors include metformin, which was tested in pre-clinical and clinical studies with conflicting results, showing either its anti-tumor effects or denying its clinical efficacy [78]. The reason for these observations might lie in the relative, but not complete, sensitivity to OXPHOS inhibitors [74].

The immune TME in malignant ascites was also characterized by transcriptomic analysis and used to construct a tumor-associated macrophage-related gene (TAMRG) prognostic signature. As expected, M1-type TAMs correlated positively with the patient’s OS, whereas M2-type TAMs, memory T CD4+ lymphocytes, neutrophils, and mast cells exhibited negative correlation, respectively. The TAMRG-based gene signature was associated with poor or favorable prognosis, depending on the gene expression profile [79].

### 2.5. Lipid Metabolism in Tumor Microenvironment

Lipid metabolism is extremely important for the growth and nutrition of peritoneal and omental implants and ovarian cancer stem cells, therefore a prognostic model based on eleven lipid metabolism gene signatures was proposed. The expression levels of several genes regulating drug-mediated and p53-mediated apoptosis, the proliferative and migratory properties of tumor cells, and the recruitment of immune cells to the tumor allowed the differentiation of patients into high- and low-risk groups [80,81,82,83,84].

### 2.6. Mechanisms of the Regulated Cell Death (RCD)

#### 2.6.1. Autophagy

Autophagy is a lysosomal-driven form of genetically determined regulated cell death (RCD). In response to multiple stressors and malnutrition, cells can control self-digestion enable survival, and maintains homeostasis. Autophagy functions as a double-edged sword, as in early cancer stages prevents tumor progression, however, in advanced tumors augments cancer stem cells and helps to survive hypoxia, drug-derived stress, and starvation. Therefore, inhibiting autophagy could be beneficial for the elimination of the tumor [85,86]. Seven autophagy-related genes have been identified as regulators of tumor immune infiltration and predictors of prognosis [87]. All the marker genes had associations with *BRCA1* and the immune pathway in ovarian cancer. Consequently, the numbers of tumor-infiltrating T, B, NK, Treg cells, neutrophils, and macrophages differed between high- and low-risk tumors. The marker genes also affect the signaling pathways, including P53-related, PI3K/AKT/mTOR, and IL-6/JAK/STAT3 pathways [87].

The study on lncRNA expression in ovarian cancer CAFs allowed the identification of several lncRNAs involved in metabolic processes and regulation of autophagy, the increase of which was correlated with a worse prognosis of patients. Another *MIR155HG* lncRNA was responsible for the regulation of genes associated with the function of the immune system, especially T cell activation, antigen presentation, cytokine signaling, and ECM interactions. Tumors with high *MIR155HG* expression had significantly higher numbers of activated T cells, M1-type macrophages, cytotoxic T CD8+ cells, and T CD4+ helper cells, and were correlated with more favorable outcomes [88].

#### 2.6.2. Ferroptosis

Except for apoptosis or autophagy, the two recently discovered forms of RCD are ferroptosis and necroptosis [89]. Ferroptosis is an iron-dependent mode of cell death, which is characterized by cytological changes in mitochondrial structure caused by the imbalance between the production and degradation of intracellular lipid reactive oxygen species. Ferroptosis eliminates malignant cells damaged by nutritional deficiency or stress [90]. In the ferroptosis-related prognostic model, the expression of genes regulating cell degradation and its recycling, defense reactions against oxidative agents, integrin-dependent signaling related to normal cell growth and tumorigenesis, the reaction of lysosomal exocytosis, prostaglandin synthesis, iron-sulfur regulation of cellular enzymes, transmembrane energy transporting, and fatty acids metabolism were correlated with patient’s OS [91]. The next study showed similarly that expression of ferroptosis-related genes including lncRNAs were correlated with prognosis in ovarian cancer patients. The prognostic model based on the 8 lncRNAs enabled the division of patients into high- and low-risk groups presented with differential clinical outcomes, mutation burden, and immune cell infiltration, and to predict the response to immunotherapy. Moreover, the expression of lncRNA RP11 correlated with the platinum sensitivity [92].

Another lncRNA signature was based on a comprehensive analysis of ferroptosis and iron metabolism-related lncRNAs (FIRLs). Patients with different FIRL signatures would be expected to respond disparately to immunotherapy. The high-risk FIRL signature differed according to the extent of infiltration by B cells, T CD4+ memory cells, NK cells, and macrophages, and high-risk FIRL scores were correlated to higher immunosuppression in the tumor environment. In this group of patients, one could anticipate a better response to immune checkpoint inhibitors-based therapy. Moreover, the low-risk FIRL signature patients were more sensitive to docetaxel, doxorubicin, etoposide, paclitaxel, cisplatin, and gemcitabine [93].

#### 2.6.3. Necroptosis

Necroptosis is regulated programmed cell necrosis, morphologically exhibiting the same features as necrosis, and mediated by pro-inflammatory cytokines (TNF-*α*, IFN-*α*, and IFN-γ), Toll-like receptors (TLR3, TLR4, and TLR9), and nucleic acid (DNA and RNA) receptors. The receptor-interacting serine/threonine kinase1 and 3 (RIPK1/3) and mixed lineage kinase domain-like pseudokinase (MLKL) are important proteins involved in the development of necroptosis [94]. Necroptosis could either indirectly enhance (via stimulation of inflammation) or directly inhibit tumorigenesis [95]. In the necroptosis-related model, the expression of genes regulating transcription activators, calcium-mediated signaling, nucleosome structure of the chromosomal fibers, activation of Janus protein kinases and death-inducing signaling pathway, initiation of an inflammatory response, and metabolism of glucose in stress conditions were identified as factors determining patient’s OS [91]. Another study devoted to necroptosis-related gene signatures identified 33 differentially expressed genes (DEGs) engaged in the regulation of necroptosis. Among genes playing a central role in the tumorigenesis and development of ovarian cancer, were those involved in TNF-*α* and NF-*ĸ*B signaling pathways [96]. Then authors constructed a risk model based on the regression analysis of a 5-gene signature which successfully differentiated patients into high or low-risk groups, with high-risk patients indicating significantly shorter survival, a decrease in expression of immune checkpoint proteins, and chemo-resistance [96].

The genomic, mutational, and epigenetic signatures of HGSTOC microenvironments are presented in Table 2.

### 2.7. Summary

In summary, the commonly known dualistic model dividing ovarian tumors into two distinct populations (type I and type II) characterized by different genetic landscapes, biology, and prognosis, has been recently found to be more complex. At least four different tumor types have been identified, namely subtypes with high stromal response, immune signature, low stromal response, and mesenchymal subtype, with low immune signature. Several studies have confirmed such division showing the presence of similar subtypes defined as proliferative, immunoreactive, differentiated, and mesenchymal tumors [17,24,25,26,27]. All other classifications more or less correspond to these four basic subtypes [32,33,37,38]. All these studies indicate, that HGSTOC cancer has different biological and clinical behaviors depending on the genetic profile and mutational burden in cancer cells, but the cellular composition of the tumor, mainly concerning the cells of both stromal and immune lineage seems to be another important determinant of tumor growth. Similarly, to cancer subtypes, the cells of the tumor microenvironment have different functional signatures which have prognostic meaning for the patients. Altogether this means, that different ovarian cancer subtypes have diverse immunological host responses, and could diversely respond to targeted therapy, therefore demanding different therapeutic approaches [37]. Moreover, diversified expression of several genes both in cancer cells themselves and in tumor microenvironment cells, could discriminate between chemo-resistant and chemo-sensitive ovarian tumors [36]. Molecular profiling indicates also that in primary HGSTOC tumors, there is a significant heterogeneity with the co-existence of several gene signatures determining its resultant phenotype and behavior, however, peritoneal cancer metastases show a reduction of heterogeneity and expansion of selected tumor cell population [38]. They also differ according to stromal and immune cell composition. That points out a potential necessity to use a different therapeutic approach to primary tumors, peritoneal implants, and recurrences.

## 3. Personalization of Treatment—State of Art and Future Directions

The attempts to get the heterogeneity of HGSTOC right have driven to the diversification of tumors into different genotypes and functional phenotypes with distinct clinical behaviors and sensitivity to drugs. Moreover, the environments of primary tumors, peritoneal implants, and ascites have been characterized by different gene signatures giving the possibility to modify the therapy according to the tumor localization and temporal changes in the course of the disease. Identification of the hub genes and their downstream pathways and targets could enable the selection of the candidate drugs for ovarian cancer treatment and improve the personalized approach to the therapy. This could change the philosophy of therapy not only in ovarian cancer but in cancer generally. An example of such an approach is an ongoing nonrandomized, multicenter phase II TAPUR clinical trial (NCT02693535) testing the use of drugs already approved by the FDA that target a specific tumor mutation in individuals with advanced cancer according to their molecular profile regardless of the tissue origin or cancer type [99]. Two study cohorts have been closed due to a lack of anti-tumor activity, but 12 cohorts have expanded to the second stage of enrollment due to promising preliminary activity. The next phase II clinical trial called NCI-MATCH (NCT02465060) attempted to answer the question of how effective treatment was based on genetic profiling in patients with solid tumors or lymphomas that had progressed despite at least one line of standard treatment [100]. The study demonstrated that next-generation sequencing in biopsy specimens from patients with relapsed-refractory cancer enables the qualification of nearly 20% of patients to evidence-based investigational therapy. However, co-occurring resistance mutations were common and that fact needs investigation of drug combination therapy regimens [101]. Another example of individualized therapy was a prospective MOSCATO 01 clinical trial. Material extracted from fresh-frozen tumor biopsies was analyzed by array comparative genomic hybridization, next-generation sequencing, and RNA sequencing. Patients were treated based on their genomic signature, and the PFS2 was compared to the PFS 1 from the recently performed therapy. An actionable molecular alteration was identified in 49% of patients and 24% of patients were treated with a targeted therapy matched to a genomic alteration. The PFS2/PFS1 ratio was >1.3 in 33% of the treated patients [102]. In the next similar trial, patients with metastatic solid tumors who had progressed on at least one line of standard-of-care therapy were referred to the Indiana University Health Precision Genomics Program. Tumor samples were studied by DNA & RNA next-generation sequencing, fluorescence in situ hybridization, and immunohistochemistry to find actionable targets. Altogether, 43% of patients treated with genomically guided therapy had a PFS ratio ≥1.3, whereas only 5% of patients treated with standard therapy had reached that result. Further, patients treated with genomically guided therapy had a superior median PFS (86 days vs. 49 days). The main limitation of the study was decision-making by a multi-disciplinary tumor board instead of an objective algorithm [103]. Another study compared the PFS in the cohort of patients with metastatic cancer of diverse subtypes who received genomic testing and targeted therapy (precision medicine), and control patients who received standard chemotherapy or best supportive care. The PFS was 23 weeks for the precision medicine group and 12 weeks for the control group, and although the inclusion of patients with supportive care could bias the results, it seemed that precision medicine may improve survival for patients with refractory cancer [104]. All these examples speak for the personalization of the treatment and implementation of drug combination therapy. Taking into consideration the aggressive course of HGSTOC personalization of the treatment is not just an option, but rather the only reasonable way and necessity.

**Figure 1:**
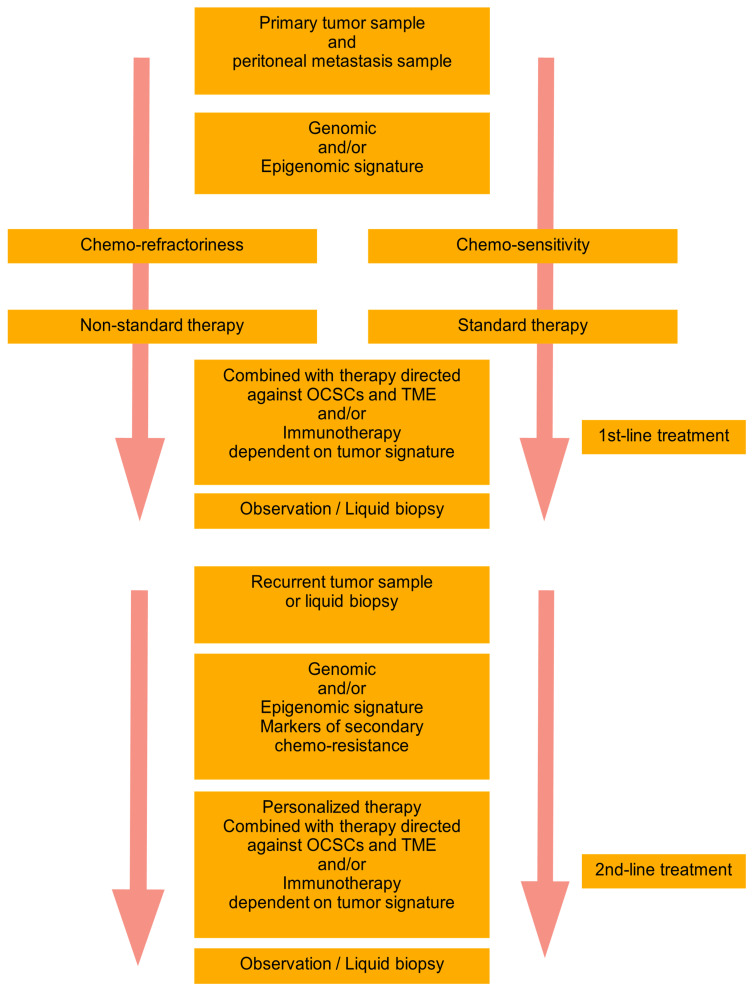
“DEPHENCE” system in ovarian cancer treatment. During primary cytoreductive surgery, the samples of the primary tumor and peritoneal metastases are collected for identification of genomic and/or epigenomic signatures. A tumor could be also sampled during exploratory laparoscopy before neoadjuvant chemotherapy. The prognosis of chemo-sensitivity or chemo-refractoriness is a base for planning the therapy. In the case of anticipated chemo-refractoriness, the non-standard therapy is started, whereas in the case of expected chemo-sensitivity, the standard chemotherapy is started. However, independently of the basic therapy, multimodal supplementary treatment is always introduced, including anti-OCSCs and TME therapy combined with immunotherapy or immune-potentization of the patient’s immune system. After the first line of treatment observation of the patient with a classical approach supplemented with repetitive liquid biopsies to monitor for response to therapy and eventually the recurrence are performed. In the case of recurrence, the tumor is again biopsied, or liquid biopsy is performed (it is a better option since ctDNA is freed from all tumor localizations, while harvest of the tissue during ordinary biopsy gives material from only one localization). Again, the genomic/epigenomic signature of the tumor is tested together with markers of chemo-resistance and appropriate personalized therapy is implemented. Similarly, to the first-line treatment, the second-line of therapy is also supplemented with anti-OCSCs and anti-TME drugs and/or immunotherapy. Due to the ovarian cancer genomic heterogeneity and temporal heterogeneity, the therapy should be tailored to the tumor genotype/phenotype and may be completely different in consecutive lines of treatment. After the second-line therapy patient is again subjected to observation and liquid biopsy, until another recurrence. OCSCs—ovarian cancer stem cells; TME—tumor microenvironment; The arrow represents Time.

Based on the recent trends and achievements in the field of HGSTOC diversity, there is a need to create a novel complex personalized model of therapy. We have postulated the basic premises of such a model called “DEPHENCE” system [10]. During primary cytoreductive surgery, the samples of the primary tumor and peritoneal metastases are collected for the identification of genomic and/or epigenomic signatures. A tumor could be also sampled during exploratory laparoscopy before neoadjuvant chemotherapy. The identification of tumor signature and prognosis of chemo-sensitivity is the base for planning the following therapy. In the case of anticipated chemo-refractoriness, the non-standard therapy is started, whereas in the case of expected chemo-sensitivity, the standard chemotherapy is started. However, independently of the basic therapy, multimodal supplementary treatment is always introduced, including anti-ovarian cancer stem cell- and tumor environment components-related therapy, combined with immunotherapy or immune-potentization of the patient’s immune system. After the first line of treatment observation of the patient with a classical approach supplemented with repetitive liquid biopsies to monitor for response to therapy and eventually the recurrence are performed. In the case of recurrence, the tumor is again biopsied, or liquid biopsy is performed (it is a better option since ctDNA is freed from all tumor localizations, while harvest of the tissue during ordinary biopsy gives material from only one localization). Again, the genomic/epigenomic signature of the tumor is tested together with markers of chemo-resistance and appropriate personalized therapy is implemented. Similarly, to the first-line treatment, the second-line of therapy is also supplemented with anti-stem cell and anti-tumor environment drugs and/or immunotherapy. Due to the ovarian cancer genomic heterogeneity and temporal heterogeneity, the therapy should be tailored to the tumor genotype/phenotype and may be completely different in consecutive lines of treatment. After the second-line therapy patient is again subjected to observation and liquid biopsy, until another recurrence (Figure 1). We have met with the opinion that such a model of therapy is non-realistic. It is true for now, but searching for the hidden order in the heterogenous HGSTOC tumors will sooner or later bring therapeutically important conclusions. Novel genetic tests and techniques are increasingly more available and cheaper. Bioinformatics enables the designing of new drugs instead of a blind search. So let us be a little more optimistic, as the history of science has taught us, that progress has many times come faster than it was expected. There is a reasonable assumption in the HGSTOC therapy to adjust the lines of treatment to the unique subtype of tumor, as well as its spatial (primary/metastatic tumor) and temporal (primary/recurrent tumor) characteristics. Without the multidirectional approach, this extremely lethal cancer will still be a step forward.

## Figures and Tables

**Table 1:** Genomic, mutational, and epigenetic signatures of HGSTOC and their meaning for prognosis and/or treatment.

Signatures of HGSTOC	Meaning	Cases [*n*]	Study Method	Reference
C1—high stromal response- high expression of stroma-regulating genes (*ACTA2*—alpha-smooth muscle actin or *α*-SMA), and moderate to extensive desmoplasia, low numbers of intratumoral CD3+ T-cells C2—high immune signature, enrichment of genes and signaling pathways associated with immune cells, markers of T-cell activation, and T-cell trafficking C4—low stromal response, high number of both intratumoral and stromal associated T CD3+ cells, and higher expression of E-cadherin C5—mesenchymal with low immune signature, over-expression of homeobox genes, high-mobility group genes, WNT/*β*-catenin and N-cadherin signaling pathways, low expression of immune response regulating genes	Patients from the C1 and C5 subtypes showed poorer survival compared with other subtypes, and the C1 subtype was especially correlated with early relapse and short OS	*n* = 285	Microarray gene expression profiling and immunohistochemistry	[17]
Type I tumors are associated with relative genetic stability and mutations of *PIK3CA*, *PTEN*, *BRAF*, *KRAS*, and *ARID1A* genes Type II tumors have chromosomal instability and defective homologous recombination repair; they are characterized mostly by *TP53*, *BRCA1*, *BRCA2*, *RB1*, and *CTNNB1* mutations	Type I tumors contain low-grade ovarian cancer (LGOC) of serous, mucinous, and endometroid histology with better prognosis and relative chemo-sensitivity Type II tumors consist of highly malignant and rapidly progressing high-grade ovarian cancer (HGOC) with poor prognosis and secondary chemo-resistance	-	Review	[18]
Mesenchymal (28%)—desmoplasia and mesenchymal invasive gene expression pattern (*HOX*, *FAP*, *ANGPTL1* genes) Immunoreactive (21%)—extensive T cell tumor infiltration and toll-like receptor signaling, expression of T-cell chemokine ligands, *CXCL11* and *CXCL10*, and the receptor, *CXCR3* Proliferative (20%)—limited inflammatory infiltration and activation of signaling pathways for stemness, high expression of *HMGA2*, *MCM2*, *PCNA*, and *SOX11*. Low expression of ovarian tumor markers (*MUC1*, *MUC16*) Differentiated (17%)—gene pattern resembling that of serous borderline tumors, high expression of *MUC16*, *MUC1*, and *SLPI*	Mesenchymal and proliferative type—unfavorable prognosis Differentiated type -intermediate prognosis Immunoreactive type—better prognosis	TCGA study *n* = 489 Konecny GE et al. *n* = 174	TCGA: Exome capture and DNA sequencing DNA copy number/genotype analysis mRNA expression profiling miRNA expression profiling CpG DNA methylation analysis Konecny GE et al.: Gene expression profiling Agilent Whole Human Genome 4 × 44 K Expression Array Irani S—Review	[24,25,26]
CLOVAR Immunoreactive CLOVAR Mesenchymal CLOVAR Proliferative CLOVAR Differentiated	The worst outcome was found in patients with CLOVAR Mesenchymal subtype resistance to chemotherapy in 63% of tumors	*n* = 489 (TCGA Biospecimens Core Resource)	Gene expression profiling Affymetrix Human Exon 1.0 ST GeneChips, Affymetrix HT-HG-U133A GeneChips Agilent 244,000 gene expression microarrays	[27]
Tumor-enriched subtype—high expression of keratin *KRT16* and *KRT23*, low expression of *PTPRC* and *PDCD1* Immune-enriched—high expression of immune *PTPRC*, *PDCD1*, *HAVCR2*, *CD274*, and low expression of *TBX21* and *NOTCH3* Mixed—a mixed expression pattern	Tumor-enriched tumors should be treated with tumor-killing therapy, while immune-enriched tumors with immunotherapy or a mixture of both approaches	*n* = 376 4188 tumor-specific genes	Whole-exome sequencing analysis Hierarchical clustering analysis Pathways enrichment analysis Analysis of the canonical markers for lineage-specific different expression genes (DEGs)	[28]
Cluster 1—over-representation of growth factor signaling pathways, Cluster 2—representation of pathways regulating cell survival in hypoxic conditions and senescence Cluster 3—related to cellular senescence	A possible treatment of choice for cluster 1 could be tyrosine kinase or angiokinase inhibitors Cluster 2 could theoretically respond to mTOR inhibitors The potential therapy for cluster 3 could be the use of deacetylase inhibitors	*n* = 450	TCGA genomic data—copy number variation, single nucleotide polymorphisms (SNPs), miRNA expression, gene expression (mRNA), DNA methylation, and clinical and outcome information	[31]
HGSTOC primary tumor—68% epithelial cells versus 11% lymphocytes HGSTOC metastatic tumor—66% lymphocytes versus 10% epithelial cells LGSOC tumor Primary tumor fibroblasts—expression of *ACTA2*, *DCN*, *ACTB* Metastatic tumor fibroblasts—*CXCL12*, *CXCL14*, *S100A6*, *S100A10*, *SFRP2*, *SFRP4*, *IGF1*, *ANGPTL4*, *IL6*, *CFB* and *SERPING1*	Compared to primary tumor-derived fibroblasts, metastatic fibroblasts were found to over-express genes regulating tumor growth, angiogenesis and inflammation	*n* = 9 Single-cell suspensions	Single-cell RNA sequencing	[32]
Epithelial cancer cells—over-expression of EpCAM, KRT5, KRT8, KRT18 and markers of fallopian tube epithelium PAX8 and KRT7 Cancer-associated fibroblasts—DCN, COL6A1, COL6A2, ACTA2), PDGFRA, PDGFRB, DDR2, FAP, and CAV1 Tumor immune cells -T and B lymphocytes, and macrophages Tumor endothelial cells—PECAM1, CDH5, and CD34 The highest activity of tumor promoters in primary tumors (NF-*κ*B, C/EBP*β)*, ETS2, HIF1, JUN). The activity of tumor promoters in both primary and metastatic tumors—JUNB, FOSL1, EGR1, ATF2, KLF13 Cluster EC1—gene enrichment for glycolysis/gluconeogenesis, and ECM-receptor interactions Cluster EC2—genes involved in the cytokine-cytokine receptor interaction, neuroactive-related pathways, and ciliated epithelial markers (FOXJ1, PIGR, CAPS, and GDF15) Cluster EC3—over-expression of genes associated with nucleotide and amino acid metabolism and function of ABC transporters Cluster EC4—characterized by the immune response-related pathways and the complement cascade Cluster EC5—gene enrichment for pathways associated with cell cycle, DNA replication, DNA repair, drug metabolism, and chemo-resistance (*FEN1*, *NEK2*, *TOP2A*)	EC5 cells could be resistant to therapy, especially with PARP inhibitors	*n* = 2 13,571 cells including epithelial cells, fibroblast cells, T cells, B cells, macrophages, and endothelial cells	Single-cell RNA sequencing	[33]
Marker genes *STAT1*, *ANP32E*, *GPRC5A*, and *EGFL6* were over-expressed Marker genes *PMP22*, *FBXO21*, and *CYB5R3* were under-expressed	Low expression of *ANP32E*, *STAT1*, *GPRC5A*, *EGFL6*, and *PMP22* was positively associated with OS Low expression of *FBXO21*, *ANP32E*, and *CYB5R3* was associated with longer PFS	*n* = 66 HGSTOC cells + data from *n* = 568 tumor samples and *n* = 7 normal ovary samples from TCGA Database	Single-cell RNA sequencing	[34]
Differentially expressed genes between ovarian cancer and controls: *KIF4A*, *KIF11*, *CDC20*, *CCNB2*, *TOP2A*, *RRM2*, *TYMS*, *BIRC5*, *BUB1B*, and *FOXM1*	*TYMS* and *BIRC5* genes were indicated as potential drug targets	*n* = 4 gene expression profiles downloaded from the Gene Expression Omnibus (GEO)	Identification of differentially expressed genes (DEGs) using GEO2R and FunRich software Functional analysis of DEGs using GO and KEGG tools Ver. 3.1.3.	[35]
Set of 8 differentially expressed genes (DEGs) as a prognostic model for survival and chemo-resistance	Up-regulation of *PNLDC1*, *VSTM2L*, *CACNA1C*, and *GDF3* related to unfavorable outcome High expression *GJA8*, *SEZ6L*, *SLC5A1*, and *SYNM* associated with better prognosis *PNLDC1* had increased expression in chemo-resistant tumors, while *SLC5A1* and *SYNM* were over-expressed in chemo-sensitive cancer	*n* = 230 TCGA OV dataset samples	mRNA expression profiling single nucleotide polymorphism (SNP), copy number variation (CNV) analysis	[36]
C1 subtype—decreased immune-cell infiltration and expression of immune checkpoint genes, the lowest incidence of *BRCA* mutation C2 subtype—up-regulated immune-cell infiltration and expression of immune checkpoint genes C3 subtype—intermediate immune status, the highest incidence of *BRCA* mutation	C1 subtype—optimal response to bevacizumab C2 subtype—displayed the worst prognosis and suboptimal response to bevacizumab C3 subtype—a secondary optimal response to bevacizumab Different ovarian cancer subtypes have different immunological host responses, could diversely respond to targeted therapy, and could demand different therapeutic approaches	*n* = 373 samples from the TCGA OV dataset *n* = 81 from the ICGC dataset *n* = 260 microarray data from GEO	Identification of ovarian cancer subtypes by non-negative matrix factorization (NMF) clustering Differential analysis and function enrichment analysis using Gene Set Enrichment Analysis (GSEA) software Gene set variation analysis (GSVA)	[37]
Quantitative Traits signatures: QT-A—genes involved in HGSTOC initiation related to cell proliferation and tumor development QT-B—genes involved in tumor-host interaction and dissemination expressed in malignant ascites, and inflammatory environment QT-C—genes involved in mesenchymal-like and non-proliferating cancer stem-like cells	QT-A signature was predominant in the proliferative HGSTOC subtype, QT-B in the inflammatory subtype, and QT-C in the mesenchymal subtype Patients having these signatures had an unfavorable outcome	*n* = 1 As11 cell line isolated from ascites and cultured	Cell proliferation assay Spheroid formation assay DNA and RNA sequencing Animal xenografting	[38]
Copy-number signatures: Signature 1—activation of RAS/MAPK signaling and telomere shortening, characterized by a low number of breakpoints per chromosome arm Signature 2—a tandem duplication through inactivation of CDK12, characterized by a high number of breakpoints and single copy-number changes Signature 3—the result of *BRCA1/2*-related homologous recombination deficiency (HRD), and was presented with the diploid and single copy changes Signature 4—whole genome duplication resulting from disturbed PI3K inactivation, characterized by multiple copy-number changes Signature 5—caused by subclonal catastrophic chromothriptic-like events of unknown origin, and characterized by subclonal copy-number changes Signature 6—focal amplification produced by loss of the cell cycle control Signature 7—caused by non-*BRCA1/2* related HRD, and characterized by different distributed copy-number changes and breaks	Signature 1—correlated with platinum-resistant relapse and poor OS Signature 2—correlated with poor OS Signature 3—correlated to favorable OS Signature 4–6—connection to survival uncertain Signature 7—correlated to favorable OS	*n* = 142 Isolated 300 DNA samples from tumor tissue *n* = 137 Germline DNA from blood samples	Tagged-amplicon sequencing Shallow whole genome sequencing (sWGS) Deep whole genome sequencing	[39]
*BRCA1/2* germ-line and somatic mutations involved about 20% of patients, while an epigenetic silencing of *BRCA1* in about 10% of patients At least 50% of HGSCs have HR pathway defects Approximately 30% of HGSC tumors have alterations in genes involved in *RB*-mediated DNA repair and cell cycle control—amplification of *CCNE1* (20% of patients), loss of *RB1* (10% of patients), or gain of *RBBP8* (4% of patients)	*BRCA1/2* germ-line and somatic mutations are represented in PARP-inhibitor-sensitive tumors *CCNE1* amplification and *BRCA1/2* loss in HGSTOC explain insensitivity to platinum and suggest that these tumors are unlikely to respond to PARP inhibitors	*n* = 6547 malignant tumor samples including *n* = 559 ovarian cancer samples	Pan-Cancer Analysis of *CCNE1* Copy Number from TCGA Copy Number Portal DNA sequencing and multiplex ligation-dependent probe amplification TCGA SNP and Gene Expression Data from Affymetrix SNP 6.0 and hthgu133a gene expression TCGA database	[43]
Disturbed expression of *SAP25*, *HLA-DPA1*, *AKT3*), and *PIK3R5*), and the mutation of *TMEM205* and *POLR2A*	Associated with the progression of chemo-resistance to paclitaxel and carboplatin	*n* = 7	Whole exome sequencing and analysis RNA sequencing and analysis Immunohistochemistry Tumor xenografting	[44]
Alterations of ERBB2 pathway members encoding genes	Combined treatment with platinum and anti-HER2 drugs showed better results	not reported	DNA and RNA next-generation sequencing DNA fingerprinting Tumor xenografting Immunohistochemistry	[45]
Seven pairs of lncRNAs had the potential to divide the population of ovarian cancer patients into high-risk and low-risk groups	High-risk scores were positively correlated with infiltration of neutrophils, macrophages, cancer-associated fibroblasts, T cells, and mast cells, and shorter OS CD244, LAG), ICOS, CTLA4, CD48, TNFRSF4M), CD80, TMIGD2, IDO1, TNFRSF18, CD274, CD40 were hypo-expressed, while CD276 and TNFRSF25 were hyper-expressed in the high-risk group	*n* = 379 ovarian cancer patients and *n* = 88 controls from TCGA and GTEx databases	RNA sequencing data Differentially expressed immune-related lncRNA (DEirlncRNA) analysis	[51]
Five differentially expressed lncRNAs differentiated HGSTOC and normal ovary	On the basis of the known function and regulatory pathways of those lncRNAs, five candidate small-molecule drugs were predicted using bioinformatics analysis	*n* = 14,087 lncRNAs were extracted from TCGA and GTEx databases	Differentially expressed immune-related lncRNA (DEirlncRNA) analysis Construction of the lncRNA-miRNA-mRNA regulatory network	[52]
DNA methylation signatures showed genes involved in integrins and cadherins signaling pathways, amino acids biosynthesis, endocrine resistance, apoptosis, focal adhesion, and cellular senescence The hypomethylated up-regulated hub genes: *TNF*, *UBC*, *SRC*, *ESR1*, *CDK1*, *PECAM1*, *CXCR4*, *MUC1*, NEMO, *IKBKG*, The hypermethylated down-regulated hub genes: *BDNF*, *CDC42*, *CD44*, *PPP2R5C*, *PTEN*, *UBB*, *BMP2*, *FOXO1*, *KLHL2*	Four genes: *TNF*, *ESR1*, *MUC1*, and *FOXO1* could function as targets for epigenetic therapy and were correlated with patients’ prognosis	*n* = 242 ovarian cancer samples and *n* = 22 normal ovaries; samples extracted from GEO gene expression datasets *n* = 10 ovarian cancer samples and *n* = 5 normal ovaries; samples extracted from GEO gene methylation datasets	Protein-protein interaction (PPI) network construction using STRING tool GO and Reactome, KEGG pathway enrichment analysis Validation of the expression of hub genes and correlations between methylation and mRNA levels using GEPIA platform	[53]
Methylation patterns identified 6 molecular clusters	Cluster 2 had the highest methylation level and the best prognosis, Clusters 4 and 5 had significantly lower methylation levels compared to the other subtypes, were connected with the HGSTOC, advanced tumors, and demonstrated very poor prognosis	*n* = 571 ovarian cancer DNA methylation samples extracted from TCGA	Clustering analysis of the methylation expression profile WGCNA coexpression analysis of CpG loci	[54]

Abbreviations: *PIK3CA*—phosphatidylinositol-4,5-bisphosphate 3-kinase catalytic subunit alpha; *PTEN*—phosphatase and tensin homolog; *BRAF*—v-Raf murine sarcoma viral oncogene homolog B; *KRAS*—Kirsten ras oncogene homolog; *ARID1A*—AT-rich interactive domain-containing protein 1A; *CTNNB1*—catenin beta 1; *HOX*—homeobox genes; *FAP*—fibroblast activation protein alpha; *ANGPTL1*—angiopoietin like 1; *HMGA2*—transcription factor high-mobility group AT-hook 2; *SOX11*—SRY-box transcription factor 11; *MCM2*—minichromosome maintenance complex component 2; *PCNA*—proliferating cell nuclear antigen; *MUC*—mucin; *CXCL11*—C-X-C motif chemokine ligand 11; *CXCL10*—C-X-C motif chemokine ligand 10; *CXCR3*—C-X-C motif chemokine receptor 3; *SLPI*—secretory leukocyte protease inhibitor; *KRT16*—keratin 16; *KRT23*—keratin 23; *PTPRC*—protein tyrosine phosphatase receptor type C; *PDCD1*—programmed cell death protein 1; *HAVCR2*—hepatitis A virus cellular receptor 2; *CD274*—programmed death-ligand 1; *TBX21*—T-box transcription factor; *NOTCH3*—neurogenic locus notch homolog protein 3; *ACTA2*—actin alpha 2; *DCN*—decorin, *ACTB*—actin beta; *CXCL12*—stromal cell-derived factor 1; *CXCL14*—chemokine (C-X-C motif) ligand 14; *S100A6–*S100 calcium-binding protein A6; *S100A10*—S100 calcium-binding protein A10; *SFRP2*—secreted frizzled-related protein 2; *SFRP4*—secreted frizzled-related protein 4; *IGF1*—insulin growth factor 1; *ANGPTL4*—angiopoietin like 1; *IL6*—interleukin-6; *CFB*—complement C3, complement factor B; *SERPING1*—serpin family G member 1; EpCAM—epithelial cell adhesion molecule; KRT5—keratin 5; KRT7—keratin 7; KRT8—keratin 8; KRT18—keratin 18; PAX8—paired box gene 8; DCN—decorin; COL6A1—collagen type VI alpha 1; COL6A2—collagen type VI alpha 2 chain ACTA2—actin alpha 2; PDGFRA—platelet-derived growth factor receptor A; PDGFRB—platelet-derived growth factor receptor B; DDR2—discoidin domain-containing receptor 2; CAV1—caveolin 1; PECAM1—platelet endothelial cell adhesion molecule 1; CDH5—VE-cadherin; CD34—transmembrane phosphoglycoprotein; *FOXJ1*—Forkhead box protein J1; *PIGR*—polymeric immunoglobulin receptor; *CAPS*—catabolite activator protein; *GDF15*—growth/differentiation factor 15; *FEN1*—flap structure-specific endonuclease 1; *NEK2*—NIMA related kinase 2; *TOP2A*—DNA topoisomerase II*α*; NF-*κ*B—nuclear factor kappa-light-chain-enhancer of activated B cells; C/EBP*β*—CCAAT/enhancer-binding protein beta; ETS2—ETS proto-oncogene 2; HIF1—hypoxia-induced factor-1; JUN—transcription factor Jun; JUNb—transcription factor Jun-B; FOSL1—Fos-related antigen 1; EGR1—early growth response 1; ATF2—cyclic AMP-dependent transcription factor; KLF13—KLF transcription factor 13; *STAT1—s*ignal transducer and activator of transcription 1; *ANP32E*—acidic leucine-rich nuclear phosphoprotein 32 family member E; *GPRC5A*—G protein-coupled receptor class C group 5 member A; *EGFL6*—EGF like domain multiple 6; *PMP22*—peripheral myelin protein 22; *FBXO21*—F-box protein 21; *CYB5R3*—cytochrome B5 reductase 3; kinesin family member 4A—*KIF4A*; kinesin family member 11—*KIF11*; cell division cycle protein 20 homolog—*CDC20*; cyclin B2—*CCNB2*, ribonucleoside-diphosphate reductase subunit M2—*RRM2*, thymidylate synthetase—*TYMS*; mitotic checkpoint serine/threonine-protein kinase BUB1 beta—*BUB1B*, forkhead box protein M1—*FOXM1*; PARN like ribonuclease domain containing exonuclease 1—*PNLDC1*; V-set and transmembrane domain-containing protein 2-like protein—*VSTM2L*; calcium voltage-gated channel subunit alpha1—*CACNA1C*; growth differentiation factor-3—*GDF3*; gap junction alpha-8 protein—*GJA8*; seizure related 6 homolog like—*SEZ6L*; sodium/glucose cotransporter 1—*SLC5A1*; synemin—*SYNM*; Sin3A associated protein 25—*SAP25*; major histocompatibility complex, class II, DP alpha 1—*HLA-DPA1*; RAC-gamma serine/threonine-protein kinase—*AKT3*; phosphoinositide 3-kinase regulatory subunit 5—*PIK3R5*; mutation of transmembrane Protein 205—*TMEM205*; polymerase (RNA) II (DNA directed)—*POLR2A*; receptor tyrosine-protein kinase erbB-2—ERBB2; natural killer cell receptor 2B4—CD244; lymphocyte-activation gene 3—LAG3; inducible T cell costimulator—ICOS; cytotoxic T cell antigen 4—CTLA4; B-lymphocyte activation marker—CD48; tumor necrosis factor receptor superfamily member 4—TNFRSF4M; T-lymphocyte activation antigen CD80—CD80; transmembrane and immunoglobulin domain containing 2—TMIGD2; indoleamine 2,3-dioxygenase 1—IDO1, TNF receptor superfamily member 18—TNFRSF18; immune inhibitory receptor ligand—CD274; type I transmembrane protein found on antigen-presenting cells—CD40; B7 homolog 3—CD276; TNF receptor superfamily member 25—TNFRSF25; tumor necrosis factor—*TNF*; ubiquitin C—*UBC*; proto-oncogene tyrosine-protein kinase Src—*SRC*; estrogen receptor 1—*ESR1*; cyclin-dependent kinase-1—*CDK1*; C-X-C motif chemokine receptor 4—*CXCR4*; NF-kappa-B essential modulator (NEMO)—*IKBKG*; brain-derived neurotrophic factor—*BDNF*; cell division control protein 42 homolog—*CDC42*; receptor for hyaluronan—*CD44*; serine/threonine-protein phosphatase 2A 56 kDa regulatory subunit gamma isoform—*PPP2R5C*; ubiquitin—*UBB*; bone morphogenetic protein 2—*BMP2*; Kelch-like family member 2—*KLHL2.*

**Table 2:** Genomic, mutational, and epigenetic signatures of HGSTOC tumor microenvironments and their meaning for prognosis and/or treatment.

Signatures	Meaning	Cases [*n*]	Study Method	Reference
Cancer-associated fibroblast subtypes: FC1—ene enrichment for lipid and steroid metabolism FC2—genes involved in glycolysis/gluconeogenesis, oxidative phosphorylation, and DNA repair pathways FC3—enriched in the genes responsible for the immune response-related pathways FC4—over-expression of angiogenesis-related genes FC5—high expression of genes regulating lipid metabolic pathways, the extracellular matrix signaling, and cellular stemness	FC2 subtype represents the highly aggressive phenotype of cancer-associated fibroblasts enhancing tumor chemo-resistance Fibroblasts from primary tumors showed high expression of the *STAR* gene, whereas those from metastatic tumors exhibited high expression of the *MFAP5* gene Interactions between tumor cells and CAFs—expression of EGFR, FGFR1, FGFR2 on the cancer cells, and ligands COPA, GRN, HB EGF, FGF2, FGF7, FGF18, on the CAF cells. Interactions EGFR-COPA and GRN/HB EGF—expressed at higher levels in metastatic compared to primary tumors	*n* = 2 13,571 cells including epithelial cells, fibroblast cells, T cells, B cells, macrophages, and endothelial cells	Single-cell RNA sequencing	[33]
The markers used for CAFs characterization were FAP, CD29, SMA, FSP1, PDGFR*β* and caveolin Cancer-associated fibroblast subsets: CAF-S1—medium/high levels of SMA, considered as “activated” CAFs CAF-S2–S3—negative/low SMA levels, defined as “non-activated” CAFs. CAF-S4—medium/high levels of SMA, considered as “activated” CAFs	HGSTOC of mesenchymal subtype, defined by stromal gene signatures and poor survival, had high numbers of CAF-S1 cells	*n* = 225 samples from the Institute Curie *n* = 285 samples from the AOCS database *n* = 484 samples from the TCGA database	Gene Expression Profiling Immunohistochemistry Primary ovarian CAF culture qRT-PCR from HGSOC and cell lines Patient-derived xenograft experiments	[55]
CAFRS index based on the expression of 4 genes—*AXL*, *GPR176*, *ITGBL1*, and *TIMP3*	A higher value of the CAFRS index indicated a higher risk of platinum-resistance	*n* = 6 50,502 ovarian cancer cells isolated Gene expression profiles extracted from GEO and UCSC Xena databases	Single-cell RNA sequencing Weighted gene co-expression network analysis	[97]
Immunological profile of HGSTOC: Activated-immune subtype CAFs-immune subtype	Activated-immune subtype showed enrichment of IFN signatures, active immune response, and better prognosis. More likely to respond to PD-1/PD-L1-based therapy CAFs-immune subtype was characterized by tumor-promoting signals like activated stroma, M2 macrophages, WNT/TGF-*β* signaling pathway, and a poor prognosis	*n* = 418 ovarian cancer samples from the TCGA database *n* = 482 samples from datasets GSE9891 and GSE32062 *n* = 431 DNA methylation samples from the UCSC Xena database	Single-sample gene set enrichment analysis (ssGSEA) Nearest Template Prediction (NTP) Tumour Immune Dysfunction and Exclusion (TIDE) Cell culture Protein Western blotting RNA qRT-PCR	[56]
Seven genes were up-regulated in HGSTOC stroma and connected with CAF activity—*PDGFRA*, *PDGFRB*, *THY1*, *PDPN*, *FAP*, *ACTA2*, *COL1A1* The CAF content is described as a CAF-score CAF scores were positively correlated with macrophages, myeloid-derived suppressor cells, neutrophils, mast cells, and with immune checkpoint regulators PD-1, PD-L1, CTLA4, LAG3, TIGIT CAF scores were negatively correlated with plasma B cells and Th1 CD4 T cells	A low CAF-score indicated a better prognosis and identified a group of patients with more immunogenic tumors which could be good candidates for immune checkpoint inhibitor therapy	*n* = 33 cancer samples from the TCGA database *n* = 31 ovarian cancer samples and *n* = 8 normal ovary samples from GEO dataset	RNA sequencing data Gene set cancer analysis (GSCA) Single-sample gene set enrichment analysis (ssGSEA) Gene set variation analysis (GSVA) enrichment Immunophenoscore (IPS) data from the The Cancer Immunome Atlas (TCIA) database	[57]
CAF-N subtype CAF-C subtype	CAF-C characterized by over-expression of INHBA-ACVR2A axis, *VCAN*, *MFAP5*, activation of Smad signaling, had more aggressive behavior and worse prognosis	*n* = 70 ovarian cancer samples	Transcriptome profiling was performed using the GeneChip Human Genome U133 Plus 2.0 microarray Hierarchical clustering analysis Crosstalk signaling pathways analysis	[58]
Cellular populations in HGSTOC ascites: - epithelial cells (*EpCAM*, cytokeratins, kallikreins +) - macrophages (*CD14*, *AIF1*, *CSF1R*, *CD163*, - CAFs (*PDPN*, decorin, *THY1*) - dendritic cells (*CD1C*, *CD1E*, *CCR7*, *CD83*) - B cells (*CD19*, *CD79A/B*) - T cells (*CD2*, *CD3D/E/G*) - erythrocytes (*GATA1*, hemoglobin). Sub-populations of CAFs with the expression of immune-related genes such as complement factors, chemokines, and cytokines (*C1QA/B/C*, *CFB*, *CXCL1*, *-2*, *-10*, *-12*, *IL6*, *IL10*) Sub-populations of macrophages with expression of M1-type markers and suppressors of M2 differentiation, and genes regulating M2 differentiation	“Immunoreactive” and “mesenchymal” subtypes of HGSTOC, reflected the abundance of immune infiltrates and fibroblasts rather than distinct subsets of malignant cells. Different populations of macrophages in malignant ascites could respond to platinum-based therapy increasing M2-phenotype	*n* = 25 ascites samples and *n* = 2 tumor samples	Plate-based single-cell RNA-sequencing Droplet-based single-cell RNA-sequencing Cell culture Spheroid formation assay Xenograft model	[61]
In several tumor clusters, the genes of fallopian tube secretory cells were over-expressed (*SOX17*, *PAX8*, *THY1*, *EpCAM*, *CRISP3*, *NR2F2*) Mesothelial cells cluster showed active EMT and activation of IL6/STAT3 signaling pathway Myofibroblasts were engaged in hypoxia-mediated EMT, active TGF-*β*-pathway CAFs were TGF-*β*-dependent and enhanced EMT Plasma cells cluster positive for *IGHG1* and *PRDM1*	This confirms the fimbrial origin of HGSTOC tumors Mesothelial cells cluster correlated to tumor growth and chemo-resistance Myofibroblasts were responsible for the promotion of metastases Cancer-associated fibroblasts promoted metastases and platinum-resistance Plasma cells were a good prognostic marker of immune active anti-tumor environment	*n* = 7 tumor samples 18,403 cell isolates from tumors	Droplet-based single-cell RNA sequencing Low-coverage whole-genome sequencing Single-cell expression profiling	[59]
Ascites spheroids contain M2-type tumor-associated macrophages and CAFs. Spheroids indicate down-regulation of angiogenesis and extra-cellular structure organization pathways and significant up-regulation of mitochondrial oxidative phosphorylation (OXPHOS) pathway	Spheroids have the characteristics typical for quiescent cells with a low proliferation profile and low chemo-sensitivity. Drugs inhibiting the OXPHOS pathway could be considered in ovarian cancer therapy	-	Review	[78]
Tumor-associated macrophage-related gene prognostic signature (TAMRG)	M1-type TAMs positively correlated with the patient’s OS M2-type TAMs, memory T CD4+ lymphocytes, neutrophils, and mast cells exhibited a negative correlation *CD163*, *VSIG4*, and *MS4A7* when over-expressed were related to poor prognosis Over-expression of *CD3E*, *IGKV4*, *TAP1* was related to favorable outcome	*n* = 6 ascites samples containing 9609 cells *n* = 375 ovarian cancer samples from the TCGA database *n* = 185 ovarian cancer samples from the GEO database	Single-cell RNA-sequencing Gene set variation analysis (GSVA) Weighted correlation network analysis	[79]
Higher expression of CXCR3/4/7 mRNA and different expression of CXCR1/2/3/4/7 mRNA in different pathological types of ovarian tumors	High CXCR7 mRNA expression and low CXCR5/6 expression were associated with unfavorable OS, while high CXCR4/7 expression and low CXCR5/6 expression were associated with a decrease in PFS The CXCR4 inhibitor was successfully used in a mouse xenograft model of ovarian cancer to restore taxol chemo-sensitivity and prolong survival	*n* = 500 cells Cancer cell line ID8	Wound healing assay Transwell invasion assay Xenograft model FACS analysis ELISA analysis	[73]
Prognostic model based on 11 lipid metabolism gene signatures engaged in drug-mediated apoptosis, proliferation and migratory properties of cancer cells, p53-mediated apoptosis, and the recruitment of immune cells to the tumor	*PI3*, *RGS1*, *ADORA3*, *CH25H*, *CCDC80*, and *PTGER3* were up-regulated in the high-risk group, whereas *KLRB1*, *CCL19*, *CXCL9* and *CXCL10* were up-regulated in the low-risk group	*n* = 9 ovarian cancer cell lines	Immunoblot analysis ELISA analysis Retrovirus-based RNA interference Proliferation and cytotoxicity assays	[80]
TRPV4 ion channel protein associated with immune pathways and with tumor-associated macrophages (TAMs) infiltrate	Expression of TRPV4 was correlated with up-regulation of immunosuppressive genes, such as regulating the function of immune checkpoints (*PD-1*, *PD-L1*, *CTLA4*, *LAG3*, *TIGIT*) and regulators of proliferation and apoptosis (*TGFB1* and *TGFBR1*), worse OS and chemo-resistance	*n* = 33 tumor samples from the TCGA dataset *n* = 33 normal ovary samples from the TCGA database *n* = 33 normal ovary samples from the GTEx database	Copy number alteration (CNA) and mutation status were analyzed using the cBioPortal database. The TIMER2 database was used to analyze the correlation between immune cell infiltration and TRPV4 ImmuCellAI database used for correlation analysis	[98]
Seven autophagy-related genes (*CAPN1*, *CDKN1B*, *DNAJB1*, *GNAI3*, *MTMR14*, *RHEB*, *SIRT2*) were identified as regulators of tumor immune infiltration and predictors of prognosis All the marker genes had associations with *BRCA1* and immune pathway in ovarian cancer	The expression *CDKN1B*, *GNAI3*, and *SIRT2* differed significantly between the high- and low-risk groups	*n* = 380 ovarian cancer samples from the TCGA database	Gene Ontology (GO), Kyoto Encyclopedia of Genes and Genomes (KEGG), and Cytoscape were used to analyze gene functions and the immune microenvironment	[87]
Analysis of ferroptosis-related genes *ATG7*, *G6PD*, *SLC3A2*, *MAP1LC3C*, *PTGS2*, *NFS1*, *VDAC2*, *ACSL3* Analysis of necroptosis-related genes *STAT5B*, *CAMK2D*, *HIST1H2AJ*, *IFNAR2*, *STAT1*, *FADD*, *CASP1*, *PYGB*, *CAMK2G*, and *HMGB1*	Over-expression of *ATG7*, *G6PD*, *SLC3A2*, *MAP1LC3C*, *PTGS2* improved OS Increased expression of *NFS1*, *VDAC2*, *ACSL3* worsened OS Expression of *STAT5B*, *CAMK2D*, *HIST1H2AJ*, *IFNAR2*, *STAT1*, *FADD* had positive role for longer OS Expression of *CASP1*, *PYGB*, *CAMK2G*, and *HMGB1* had bad influence on OS	*n* = 260 ovarian cancer samples of platinum-treated tumors extracted from the GSE32062 database *n* = 185 ovarian cancer samples of platinum-treated tumors extracted from the GSE26712 database *n* = 110 ovarian cancer samples of platinum-treated tumors extracted from the GSE17260 database	Gene enrichment and microenvironment analyses using the limma package and GSVA software Ver. 1.32.0. were used to compare the high- and low-risk ovarian cancer patients	[91]
Ferroptosis-related signature of 8–11 lncRNAs (RP11-443B7.3, RP5-1028K7.2, TRAM2-AS1, AC073283.4, RP11-486G15.2, RP11- 95H3.1, RP11-958F21.1, and AC006129.1)	Divided patients into high- and low-risk groups presented with differential clinical outcomes, immune cell infiltration, platinum-sensitivity, and predicted response to immunotherapy	*n* = 357 patients Gene expression profile data and clinical follow-up were extracted from the TCGA database	Heterogeneous Clustering Analysis Gene Set Enrichment Analysis (GSEA) Risk scoring model for ferroptosis-related lncRNAs	[92]
Necroptosis-related hub genes playing a central role in the ovarian tumorigenesis: *CASP8*, *CDKN2A*, *CFLAR*, *CYLD*, *DDX58* and *DIABLO* Differentially expressed 5 genes (DEGs) Introduced to the prognostic model (*UBD*, *ISG20*, CXCL*11*, *ATP1A3*, and *HLA-DOB*)	The model differentiates patients into high or low-risk groups, with high-risk patients indicating significantly shorter survival and chemo-resistance	*n* = 587 patients RNA-seq data, mutation data, and clinical information extracted from the TCGA database *n* = 260 patients RNA-seq data, mutation data, and clinical information extracted from the GTEx database	NMF Consensus Clustering Development and validation of the necroptosis-related prognostic signature Gene Ontology (GO) and Kyoto Encyclopedia of Genes and Genomes (KEGG) functional enrichment analyses	[96]
lncRNAs engaged in metabolic processes and autophagy regulation	Up-regulation of *DANCR*, *LOC642852*, *MALAT1*, *MEG3*, *MGC2752*, *TP73-AS1*, and *XIST* lncRNAs was correlated to the worse outcome Tumors with high lncRNA *MIR155HG* expression had significantly higher numbers of activated T cells, M1-type macrophages, cytotoxic T CD8+ cells, and T CD4+ helper cells, and were correlated with a more favorable outcome	*n* = 67 ovarian cancer samples	RNA isolation, amplification, and hybridization to GeneChip Human Genome U133 Plus 2.0 Oligonucleotide arrays Functional prediction of lncRNAs associated with patient survival CIBERSORT analysis	[88]
Immune-related lncRNA pairing model based on 7 lncRNAs pairs: USP30-AS1-AC008649.2, AC007389.5-AC073046.1, AC005884.2-AL163051.1, U62317.1-HOXB-AS2, BMPR1B-DT-UNC5B-AS1, AL035701.1-AC106900.1, NR4A1AS-LINC00893	The high-risk lncRNA signatures correlated with tumor infiltration with macrophages, neutrophils, T and mast cells, CAFs, and low expression of immune checkpoint genes	*n* = 379 ovarian cancer patients and *n* = 88 controls from TCGA and GTEx databases	RNA sequencing data Differentially expressed immune-related lncRNA (DEirlncRNA) analysis	[51]
Eight ferroptosis and iron metabolism-related lncRNAs (FIRLs): AC138904.1, AP005205.2, AC007114.1, LINC00665, UBXN10-AS1 AC083880.1, LINC01558, and AL023583.1	The high-risk FIRLs signature correlated to higher immunosuppression in the tumor environment. In this group of patients, one could anticipate a better response to immune checkpoint inhibitors-based therapy The low-risk FIRLs signature patients were more sensitive to docetaxel, doxorubicin, etoposide, paclitaxel, cisplatin, and gemcitabine	*n* = 374 patients Clinical data, RNA sequencing profiles, and normal ovarian tissue RNA sequencing profiles extracted from TCGA and GTEx databases	Pearson correlation between ferroptosis and iron-metabolism-related genes Cox-Lasso regression analysis ROC curve, Kaplan–Meier analysis, decision curve analysis (DCA), Cox regression analysis and calibration curve	[93]

Abbreviations: *STAR—s*teroidogenic acute regulatory protein; *MFAP5*—microfibril associated protein 5; EGFR—epithelial growth factor receptor; FGFR1—fibroblast growth factor receptor1; FGFR2—fibroblast growth factor receptor2; COPA—coatomer subunit alpha; GRN—granulin precursor; HB EGF—heparin-binding EGF-like growth factor; FGF2—fibroblast growth factor 2; FGF7—fibroblast growth factor 7; FGF18—fibroblast growth factor 18; FAP—fibroblast activation protein alpha; CD29—integrin-*β*1; SMA—smooth muscle *α*-actin; FSP1—fibroblast-specific protein 1; PDGFR*β*—platelet-derived growth factor receptor A; *AXL*—tyrosine-protein kinase receptor UFO; *GPR176*—G-protein coupled receptor 176; *ITGBL1*—integrin subunit beta like 1; *TIMP3*—TIMP metalloproteinase inhibitor 3; WNT—Wingless and Int-1; TGF—transforming growth factor; PD-1—programmed death 1; PD-L1—programmed death 1 ligand; LAG3—lymphocyte-activation gene 3; TIGIT—T cell immunoreceptor with Ig and ITIM domains; INHBA—inhibit beta A; ACVR2A—activin receptor type-2A; VCAN—versican; *MFAP5*—microfibrillar-associated protein 5; Smad—serine/threonine kinase receptor transmitting TGF-beta superfamily member’s signals; CD14—cluster of differentiation 14; AIF1—allograft inflammatory factor 1; CSF1R—colony stimulating factor-1 receptor; CD163—high affinity scavenger receptor for the hemoglobin-haptoglobin complex; PDPN—podoplanin; DCN—decorin; THY1—T-cell marker; CD1C—marker for Langerhans cells; CD1E—T-cell surface glycoprotein; CCR7—C-C chemokine receptor type 7; CCR7—marker for mature dendritic cells; CD19—cluster of differentiation 19; CD79A/B—B-cell antigen receptor complex-associated protein alpha/beta chains; CD2—cluster of differentiation 2; CD3D/E/G—T-cell surface glycoprotein CD3; GATA1—GATA-binding factor 1; *C1QA/B/C*—complement components 1q; *CFB*—complement factor B; *CXCL1*, *-2*, *-10*, *-12*—C-X-C motif chemokines 1, -2, -10, -12; *IL6*—interleukin 6; *IL10*—interleukin 10; *SOX17*—SRY-Box Transcription Factor 17; *PAX8*—Paired Box 8; *CRISP3*—cysteine-rich secretory protein 3; *NR2F2*—nuclear receptor subfamily 2 group F member 2; *IGHG1*—immunoglobulin Heavy Constant Gamma 1; *PRDM1*—PR/SET Domain 1; *CD163*—Cluster of Differentiation 163; *VSIG4*—V-set and immunoglobulin domain containing 4; *MS4A7*—membrane-spanning 4-domains subfamily A member 7; *IGKV4*—immunoglobulin kappa variable 4; *TAP1*—transporter associated with antigen processing 1; *PI3*—peptidase inhibitor 3; *RGS1*—regulator of G-protein signaling 1; *ADORA3*—adenosine A3 receptor—*ADORA3*; *CH25H*—cholesterol 25-hydroxylase; *CCDC80*—coiled-coil domain-containing protein 80; *PTGER3*—prostaglandin E2 receptor EP3 subtype; *KLRB1*—killer cell lectin-like receptor subfamily B, member 1; *CCL19*—C-C motif chemokine ligand 19; *CXCL9*—C-X-C motif chemokine ligand 9; *CXCL10*—C-X-C motif chemokine ligand 10; *TGFB1*—transforming growth factor TGF-*β*1; *TGFBR1*—TGF-*β*1 receptor; *CAPN1*—calpain-1 catalytic subunit; *CDKN1B*—cyclin-dependent kinase inhibitor 1B; *DNAJB1*—DnaJ homolog subfamily B member 1; *GNAI3*—guanine nucleotide-binding protein G(k) subunit alpha; *MTMR14*—myotubularin related protein 14; *RHEB*—Ras homolog enriched in brain; *SIRT2*—sirtuin 2; *ATG7*—autophagy-related 7; *G6PD*—glucose-6-phosphate dehydrogenase; *SLC3A2*—solute carrier family 3 member 2; *MAP1LC3C*—microtubule-associated proteins 1A/1B light chain 3B; *PTGS2*—cyclooxyrgenase 2; *NFS1*—mitochondrial cysteine desulfurase; *VDAC2*—voltage-dependent anion-selective channel protein 2; *ACSL3*—long-chain-fatty-acid—CoA ligase 3; *STAT5B*—signal transducer and activator of transcription 5B; *CAMK2D*—calcium/calmodulin-dependent protein kinase type II delta chain; *HIST1H2AJ*—histone H2A type 1-J; *IFNAR2*—interferon-alpha/beta receptor beta chain; *STAT1*—signal transducer and activator of transcription 1; *FADD*—Fas-associated protein with death domain; *CASP1*—caspase-1/interleukin-1 converting enzyme; *PYGB*—glycogen phosphorylase, brain—*PYGB*; *CAMK2G*—calcium/calmodulin-dependent protein kinase type II gamma chain; *HMGB1*—high-mobility group protein 1; *CASP8*—caspase 8; *CDKN2A*—cyclin dependent kinase inhibitor 2A; *CFLAR*—CASP8 and FADD like apoptosis regulator; *CYLD*—CYLD lysine 63 deubiquitinase; *DDX58*—DExD/H-box helicase 58; *DIABLO*—diablo IAP-binding mitochondrial protein; *UBD*—ubiquitin D; *ISG20*—interferon-stimulated gene of 20 kDa protein; *BATF2*—basic leucine zipper ATF-like transcription factor 2; *CXCL11*—C-X-C motif chemokine ligand 11; *HLA-DOB*—major histocompatibility complex, class II, DO beta; *ATP1A3*—ATPase Na^+^/K^+^ transporting subunit alpha 3.
